# CHANGES IN OLDER ADULTS’ PERCEPTIONS OF AGE-FRIENDLINESS IN HONG KONG: A THREE-YEAR MIXED-METHODS STUDY

**DOI:** 10.1093/geroni/igae098.1329

**Published:** 2024-12-31

**Authors:** Terry Lum, Hiu-kwan Chui, Shiyu Lu, On Fung Chan, Yingqi Guo, Jennifer Tang, Yuqi Liu

**Affiliations:** The University of Hong Kong, Hong Kong, China (People’s Republic); The University of Hong Kong, Hong Kong, China (People’s Republic); The University of Hong Kong, Hong Kong, China (People’s Republic); The University of Hong Kong, Hong Kong, China (People’s Republic); Hong Kong Baptist University, Kowloon Tong, Hong Kong; Chinese University of Hong Kong, Hong Kong, China (People’s Republic); South China University of Technology, Guangzhou, Guangdong, China (People’s Republic)

## Abstract

Hong Kong is known for its high population density, high urbanization, and hilly terrain, making age-friendliness a challenge in the city. Yet, the city is aging rapidly; by 2041, one in three residents will be 65 and older. Although age-friendly city has become a significant public policy imperative in recent decades, there is a dearth of empirical evidence on how AFC initiatives can improve perceived age-friendliness among community-dwelling older adults and how such initiatives may affect older adults with different socioeconomic statuses differently. Drawing on a three-year citywide AFC initiative in Hong Kong, we conducted a trend study to evaluate changes in perceived age-friendliness in eight AFC domains with 2575 and 2697 community-dwelling older adults in 2015 and 2018 respectively, in addition to 36 focus groups involving 206 older adults. Participants were asked to share their views on changes in age-friendliness in their cities. Survey data were analyzed using linear regression, while focus group data were analyzed using thematic analysis. Significant improvements were found in perceived age-friendliness in all eight AFC domains. Low-income older adults saw the greatest improvements in age-friendliness. Thematic analysis revealed that despite improvements, shortcomings persist in housing, civic engagement, and employment domains. Nevertheless, our findings demonstrate that concerted efforts can improve a city’s overall age-friendliness and that such improvements appear most evident among low-income older adults.

